# The LOX-1 Scavenger Receptor and Its Implications in the Treatment of Vascular Disease

**DOI:** 10.1155/2012/632408

**Published:** 2012-02-19

**Authors:** M. W Twigg, K. Freestone, S. Homer-Vanniasinkam, S. Ponnambalam

**Affiliations:** ^1^Endothelial Cell Biology Unit, Institute of Molecular & Cellular Biology, LIGHT Laboratories, Clarendon Way, Leeds LS2 9JT, UK; ^2^Leeds Vascular Institute, Leeds General Infirmary, Great George Street, Leeds LS1 3EX, UK

## Abstract

Cardiovascular disease is the leading cause of death. The disease is due to atherosclerosis which is characterized by lipid and fat accumulation in arterial blood vessel walls. A key causative event is the accumulation of oxidised low density lipoprotein particles within vascular cells, and this is mediated by scavenger receptors. One such molecule is the LOX-1 scavenger receptor that is expressed on endothelial, vascular smooth muscle, and lymphoid cells including macrophages. LOX-1 interaction with OxLDL particles stimulates atherosclerosis. LOX-1 mediates OxLDL endocytosis via a clathrin-independent internalization pathway. Transgenic animal model studies show that LOX-1 plays a significant role in atherosclerotic plaque initiation and progression. Administration of LOX-1 antibodies in cellular and animal models suggest that such intervention inhibits atherosclerosis. Antiatherogenic strategies that target LOX-1 function using gene therapy or small molecule inhibitors would be new ways to address the increasing incidence of vascular disease in many countries.

## 1. Background

Cholesterol is a key cellular molecule that is vital to growth and repair and is also a major component of the mammalian plasma membrane bilayer. It is also required for the synthesis of steroid hormones and bile salts. However, an elevated level of serum cholesterol and in particular, the low-density lipoprotein (LDL) fraction are well-established risk factors for the development of atherosclerosis. Atherosclerosis is a systemic disease, commonly affecting multiple vascular beds [1] and is the leading cause of death in Europe [2]. Uptake of native LDL into cells was first proposed to be a crucial initial step in the pathogenesis of atherosclerosis with the discovery of the LDL receptor in the 1970's by Goldstein and Brown [3]. Their studies led to the elucidation of a pathway by which the LDL-receptor complex undergoes receptor-mediated endocytosis to enable it to be taken into the cell. They observed that individuals with homozygous familial hypercholesterolaemia (FH), who suffer with manifestations of atherosclerosis in their teens, are completely lacking in functional LDL receptors, and, therefore, their circulating cholesterol levels are up to 5 times higher than in normal individuals. However, this did not explain the observation that patients with FH accumulated cholesterol in their cells despite the lack of functional LDL receptors. Furthermore, lipid-laden foam cells derived from patient tissues did not develop *in vitro* even in the presence of high concentrations of native LDL in contrast to controls. Their work led to the now widely accepted view that the internalisation of a modified form of LDL rather than native LDL is responsible for the development of atherosclerotic plaque, and that this occurs via membrane-bound receptors distinct to that of the native LDL receptor. Basu et al. first demonstrated this in an experiment in which an iodine-labelled acetylated form of LDL could induce massively increased cholesterol accumulation in macrophages *in vitro *[4]. Further work *in vitro* has shown that the oxidative modification of LDL increases the cellular content of cholesterol. Today, several receptors have been identified that bind and internalise oxidised LDL (OxLDL). This review focuses on one of those receptors, the lectin-like oxidised low-density lipoprotein receptor-1 (LOX-1) and explores its potential as a therapeutic target.

## 2. Modification of LDL

The oxidative modification of LDL results in a particle with greatly increased proatherogenic and proinflammatory properties than that of native LDL. It also becomes a ligand for scavenger receptors but is not recognised by the native LDL receptor, thought to be due to the greater net negative charge [5]. The “average” LDL particle has been calculated to contain 600 molecules of free cholesterol, 1600 molecules of cholesteryl ester, 700 molecules of phospholipid, 180 molecules of triglyceride, and 1 molecule of apolipoprotein B-100 [6]. All of these molecules can undergo oxidative damage, and, therefore, understandably there is a spectrum of oxidation level to the OxLDL created experimentally. It is believed that the amount of OxLDL in the human circulation is negligible, due to the presence of antioxidants. OxLDL has, however, been extracted from human and rabbit atherosclerotic plaque, in a form recognisable by scavenger receptors [7]. The oxidation is likely to take place in pockets within the subendothelial layer, where the cells produce both free radical and nonradical oxidants, and the relative concentration of antioxidants is lower. The resulting OxLDL has an immediate proatherogenic effect; it causes the migration of monocytes to the area via stimulation of the release of monocyte chemoattractant protein-1 [8] and promotes the differentiation of monocytes to macrophages by stimulating the release of macrophage-colony stimulating factor from endothelial cells [9]. OxLDL is cytotoxic to endothelial cells *in vitro* and inhibits the vasodilation normally induced by nitric oxide [10].

## 3. LOX-1

Modified LDL is internalized or endocytosed by membrane-bound scavenger receptors, and as it stands today, numerous scavenger receptors have been identified that recognize OxLDL. Scavenger receptors are membrane-bound proteins that are capable of binding a wide variety of ligands. The scavenger receptor family is subdivided into 8 subclasses. The class A scavenger receptors all bind modified LDL and are primarily expressed on macrophages. Class B includes the CD36 receptor, which has been shown to bind and internalize modified LDL [11]. The affinity of the other classes to bind modified LDL is less well described, except for the class E receptor, lectin-like OxLDL receptor-1 (LOX-1). 

LOX-1 (Genbank designation OLR1) was first cloned as a major receptor for OxLDL in 1997 by Sawamura and colleagues [12]. LOX-1 is expressed on a variety of cell types, including endothelial cells, platelets, macrophages, and smooth muscle cells [13–15]. The human ortholog is a 50 kDa type II transmembrane glycoprotein comprising 273 amino acids [12]. The mammalian protein is comprised of a short N-terminus cytoplasmic domain, transmembrane domain, neck domain, and a C-type lectin-like domain (CTLD). To function, LOX-1 requires oligomerisation of a homodimer; this basal complex contains 2 LOX-1 polypeptides linked via an intermolecular disulfide bond in the neck domain via residue C140 [16]. Evidence increasingly suggests that LOX-1 activity promotes vascular dysfunction and atherosclerosis. LOX-1 ablation in transgenic murine models reduces atherosclerotic plaque development [17] whereas overexpression of LOX-1 in apolipoprotein E-null mice increases atheroma-like lesions 10-fold [18]. Interestingly, raised serum levels of a soluble LOX-1-derived proteolytic fragment correlate with elevated acute coronary syndromes (ACS) [19] and type II diabetes [20]. To date, seven single nucleotide polymorphisms (SNP) within the LOX-1 gene have been found (six noncoding, one coding) [21]. One SNP appears to confer protection against OxLDL-induced macrophage apoptosis [22], whereas the K167N polymorphism, resulting in a lysine to asparagine substitution, increases the risk of myocardial infarction in a specific patient cohort [23]. Numerous signal transduction pathways are associated with LOX-1 activation by OxLDL binding including RhoA/Rac1, p38MAPK, protein kinase B and C, and ERK1/2, and blocking LOX-1 function in primary endothelial cells inhibits proinflammatory signalling, NF-*κ*B activation, and apoptosis [24, 25]. 

### 3.1. Plasma Membrane Endocytosis of LOX-1

**Figure 1:**
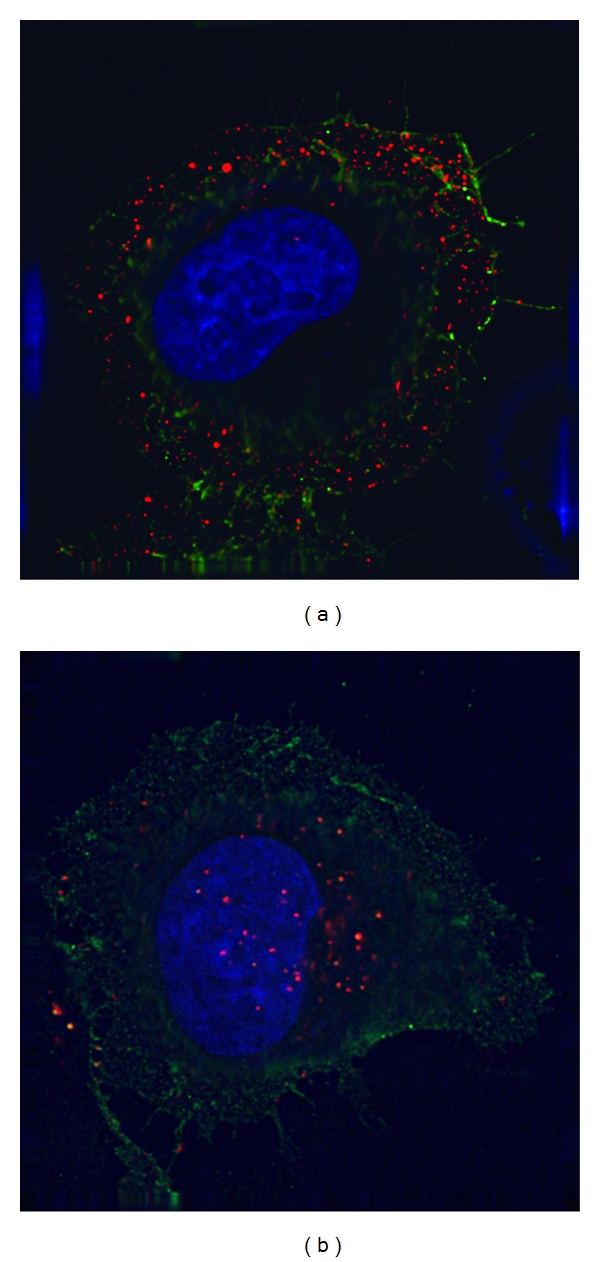
Internalisation of OxLDL. Epithelial HeLa cells transiently expressing a LOX-1-FLAG protein (green) were incubated with 10 *μ*g/mL DiI-OxLDL (red) for 5 mins at 37°C (pulse) to allow binding (a), and chased for a further 55 mins to allow internalization (b) before fixation. Nuclei were stained with DAPI (blue). Specimens were visualized using a deconvolution microscope using previously described procedures [26].

OxLDL is rapidly internalised into cells upon binding LOX-1 and can be observed in punctate perinuclear structures less than an hour after exposure to a LOX-1 expressing cell (Figure 1). 

This internalisation is blocked by the LOX-1-blocking-antibody JTX92 [27], which prevents binding of OxLDL. LOX-1 mediates early steps in internalisation of OxLDL but within an hour of internalisation is mostly uncoupled from OxLDL, and both molecules are located in separate subcellular compartments within the cytosol [26]. This internalisation pathway is not dependant on LOX-1 binding to OxLDL as the receptor is constitutively endocytosed from the plasma membrane. 

**Figure 2:**
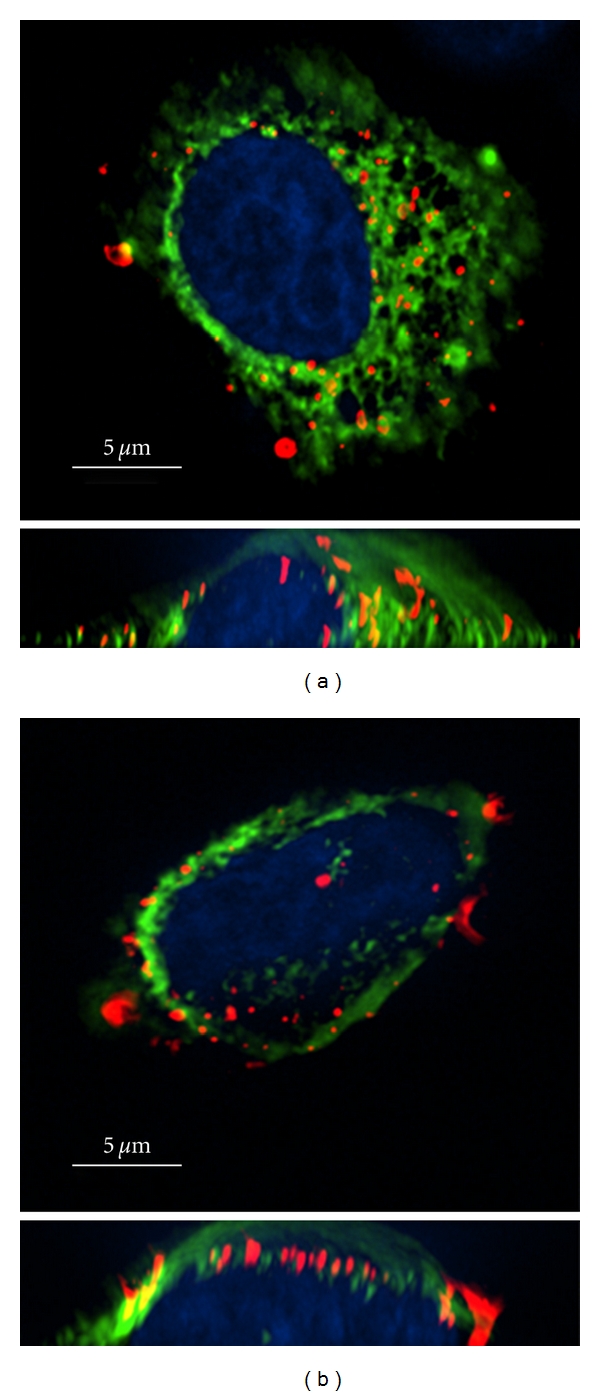
Dynamin-2 regulates OxLDL endocytosis. Epithelial HeLa cells expressing both LOX-1-FLAG and either (a) wild-type dynamin 2 (green) or (b) a dynamin-2 K44A mutant (green) were incubated and chased with labelled Dil-OxLDL (red). Cells were fixed and processed for fluorescence microscopy using previously described procedures [26]. The nuclei were stained with DAPI (blue). Bar, 5 *μ*g/m. A slice through the cell is shown in the top half of the panel and a cross-section through the cell is shown at the bottom.

The main form of receptor-mediated endocytosis is clathrin-mediated endocytosis, but it has become apparent that clathrin-independent pathways may represent up to 50% of cellular uptake [28]. Reverse genetic experiments have shown that both the clathrin heavy chain and the AP-2 adaptor complex are not required for endocytosis of LOX-1 [26]. LOX-1 was also shown not to colocalise with the caveolae marker caveolin-1. However, there is evidence that caveolae may play a part in the endocytosis of OxLDL in an endothelial model [29]. The dynamin-2 GTPase has been shown to be essential for LOX-1 endocytosis as expression of a dominant-negative protein defective in GTPase activity blocked OxLDL uptake via the LOX-1 scavenger receptor (Figure 2). The full mechanism by which LOX-1 endocytosis occurs has yet to be established.

In eukaryote cells, receptor-mediated endocytosis is regulated by the recognition of cytoplasmic motifs by cellular machinery which promotes the selection of “cargo” for transport within transport intermediates such as membrane-bound vesicles [30]. The cytoplasmic domain of LOX-1 does not contain any previously characterised motifs such as the tyrosine-based motif YxxΦ  or the di-leucine-based motifs. Alanine-scanning mutagenesis of the LOX-1 cytoplasmic domain allowed the identification of a sequence of three contiguous residues (DDL) at position +4 to +6 which regulate LOX-1 endocytosis [31]. This diacidic DDL motif thus defines a new class of novel endocytic motifs that mediate clathrin- and AP2-independent endocytosis at the plasma membrane. This motif is transplantable, as replacement of the transferrin receptors cytoplasmic domain with the LOX-1 cytoplasmic domain still promotes constitutive endocytosis. Endocytosis of this transferrin receptor-LOX-1 protein chimera was blocked by replacement of the DDL motif with a triple alanine sequence, thus showing specificity in this endocytosis [31]. 

After internalization at the plasma membrane, LOX-1 has been shown to be separated from OxLDL in endosomes [27]. Immunofluorescence studies demonstrate that after 15 min of internalization LOX-1 and OxLDL are colocalised in the same endocytic compartment. From 30 min onwards after ligand binding, this codistribution disappears, indicating that LOX-1 and OxLDL have now been sorted into separate compartments. OxLDL is trafficked through the early endosome and on to the lysosome for degradation. It is believed that the majority of LOX-1 is targeted back to the plasma membrane by a recycling pathway from endosome-to-plasma membrane. Cytosolic factors that mediate recognition, endocytosis, and/or recycling of LOX-1 have yet to be identified. Targeting such factors using genetic or pharmacological approaches would be a potential means of blocking the proatherogenic function of LOX-1 in promoting atherosclerosis.

LOX-1 appears to be essential for the phagocytosis of aged and apoptotic cells in endothelial cells [32]. LOX-1 was demonstrated to be necessary for phagocytosis of such cellular bodies in transfected Chinese hamster ovary cells, but this is blocked by OxLDL indicating a competition between OxLDL and apoptotic/aged bodies for binding to the same or adjacent site on LOX-1. Phosphatidylserine (PS) recognition on the plasma membrane of aged/apoptotic cells has been shown to be important in their phagocytosis [33], and LOX-1 is able to recognise PS on apoptotic cells in a calcium-dependent manner [34]. 

LOX-1 endocytosis is also potentially important in immune surveillance as it has been shown to regulate antigen presentation by MHC class I and II molecules on dendritic cells [35] and B cells [36]. LOX-1 is also essential for the endocytosis of various heat shock proteins (hsp's) complexed to antigen-derived peptides [37]. LOX-1 is postulated to deliver the antigenic complex to endosome-like compartments, where it is presumably loaded onto MHC molecules, which mediate antigen presentation to CD4+ and CD8+ T cells. 

## 4. Conclusion

**Figure 3:**
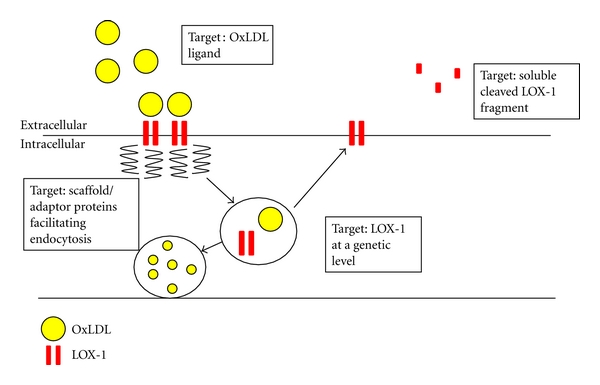
Schematic diagram showing potential routes towards blocking OxLDL and/or LOX-1 function and trafficking to attenuate the pathological process of atherosclerosis.

Scavenger receptors are heavily implicated in the process of atherosclerotic plaque formation [38]. This review focuses on LOX-1; however, there is similar evidence for the involvement of several other classes of scavenger receptor. For example, ApoE-null (−/−) mice lacking macrophage CD36 had an 88% decrease in atherosclerotic lesion area in the aorta compared to controls [39], despite the heterogeneity amongst the scavenger receptor family and their multiligand capabilities, their targeting could prove fruitful in the moderation of vascular disease, especially considering the limited success of current treatment modalities. However, the most effective means of targeting and disrupting the OxLDL- LOX-1 endocytic process remains unclear (Figure 3). The OxLDL ligand itself could be targeted; in a murine model, LOX-1 expressed ectopically in the liver via adenovirus administration reduced levels of circulating OxLDL and inhibited the formation of atherosclerotic lesions [40], gene therapy with possible genomic manipulation of scavenger receptor expression by delivery of transgenes or by blockade of gene expression may be possible [41]. Further understanding of the proteins that facilitate OxLDL transfer across the endothelium may allow the development of pharmaceutical agents that inhibit its endocytosis. Finally, the role of the soluble fragment of the LOX-1 receptor that is shed into the circulation is unclear, but the study of its use as a biomarker in the treatment of cardiovascular disease is promising. 

Significant recent progress has been made in the elucidation of the pathways of internalization of the OxLDL-LOX-1 complex; however, there remains much to be done to further characterise the role of this multiligand scavenger receptor in regulating human health and disease. The existence of several different subclasses of scavenger receptor capable of internalising OxLDL makes the development of targeted therapies a more complex issue. The molecular study of scavenger receptors remains an exciting avenue in the search for therapeutic agents to attenuate the atherosclerotic process in humans.
